# Responses of photosynthetic capacity to soil moisture gradient in perennial rhizome grass and perennial bunchgrass

**DOI:** 10.1186/1471-2229-11-21

**Published:** 2011-01-25

**Authors:** Zhenzhu Xu, Guangsheng Zhou

**Affiliations:** 1 State Key Laboratory of Vegetation and Environmental Change, Institute of Botany, Chinese Academy of Sciences; 20 Nanxincun, Xiangshan, Haidian, Beijing 100093, PR China; 2 Chinese Academy of Meteorological Sciences, 46 Zhongguancun Nandajie, Haidian, Beijing 100081, PR China

## Abstract

**Background:**

Changing water condition represents a dramatic impact on global terrestrial ecosystem productivity, mainly by limiting plant functions, including growth and photosynthesis, particularly in arid and semiarid areas. However, responses of the potential photosynthetic capacity to soil water status in a wide range of soil moisture levels, and determination of their thresholds are poorly understood. This study examined the response patterns of plant photosynthetic capacity and their thresholds to a soil moisture gradient in a perennial rhizome grass, *Leymus chinensis*, and a perennial bunchgrass, *Stipa grandis*, both dominant in the Eurasian Steppe.

**Results:**

Severe water deficit produced negative effects on light-saturated net CO_2 _assimilation rate (*A*_sat_), stomatal conductance (*g*_s_), mesophyll conductance (*g*_m_), maximum carboxylation velocity (*V*_c,max_), and maximal efficiency of PSII photochemistry (*F*_v_/*F*_m_). Photosynthetic activity was enhanced under moderate soil moisture with reductions under both severe water deficit and excessive water conditions, which may represent the response patterns of plant growth and photosynthetic capacity to the soil water gradient. Our results also showed that *S*. *grandis *had lower productivity and photosynthetic potentials under moderate water status, although it demonstrated generally similar relationship patterns between photosynthetic potentials and water status relative to *L. chinensis*.

**Conclusions:**

The experiments tested and confirmed the hypothesis that responsive threshold points appear when plants are exposed to a broad water status range, with different responses between the two key species. It is suggested that vegetation structure and function may be shifted when a turning point of soil moisture occurs, which translates to terms of future climatic change prediction in semiarid grasslands.

## Background

Water shortage constrains terrestrial ecosystem productivity considerably, mainly by limiting vegetation structure, such as species components, and their functions including growth and photosynthesis, particularly in arid and semiarid areas with large spatial-temporal variances [1-4]. Drought has been and is becoming a most critical issue under climate change, with the temperature expected to increase continually, further enhancing drought severity by accelerating evapotranspiration of the ecosystems [5-7]. The grassland of North China covers 41% of the total land area of China. However, approximately 90% of the grassland nationally has degraded to various extents during recent decades due to intensified land use, e.g., overgrazing and improper reclamation, and adverse climatic change, e.g., water deficit [2,8,9]. Water scarcity accompanying rising temperature could become an increasing environmental concern in this grassland ecosystem, leading to a reduction in productivity and negative alterations in the ecosystem structure and carbon balance, with consequent severe deterioration [5,10-12].

Many studies have indicated that mild drought has no obvious impact on plant growth and photosynthesis, even stimulation to a certain degree, but severe drought can lead to dramatic reductions [3,13,14]. Whether photosynthetic capacity completely recovers after rewatering depends on the drought-resistance of different species [3,15-17]. Most experimental results have shown that the net photosynthetic rate (*A*), stomatal conductance (*g*_s_), and mesophyll conductance (*g*_m_) generally decrease with decreasing water availability, and an obvious reduction in photosynthesis occurs due to severe water deficit. Reduction in net CO_2 _assimilation can be attributed to both stomatal and biochemical limitations, in which the proportional contribution of the latter may increase with drought severity [18-22]. Parry et al. [23] reported that in tobacco plants, Rubisco activity, a key photosynthetic activity marker, gradually decreases with decreasing relative water content. However, in many studies, photosynthetic potentials are not significantly affected by moderate water deficit, and only under severe drought does obvious inhibition arise. Dissimilarities may be related to drought treatment duration, as well as differing responses from species [24,25].

The central parameters representing photosynthetic capacity, including light-saturated net CO_2 _assimilation rate (*A*_sat_), maximum *in vivo *carboxylation velocity (*V*_c,max_), and maximum rate of electron transport (*J*_max_) have been used to assess the effects of global changes on the biosphere, although parameters may be altered under different growth conditions [26,27]. Unfortunately, the parameterization of responses of the photosynthetic capacity to broad ranges of soil water status has received relatively scant focus to date, although a number of studies with one or a few drought intensifications have been conducted. The lack of relatively accurate parameters of environmental response curves for plant biomass and photosynthetic capacity might have substantially limited the proper application of stimulation models on plant productivity [26-28].

Many studies have suggested a simple linear relationship of plant biomass with water status or a precipitation gradient in arid/semiarid regions [29,30]. In contrast, at excessive moisture level, photosynthesis and stomatal conductance, as well as carbohydrate partitioning also might be constrained due to hypoxia in the rhizosphere [31-34]. Larger rainfall events have also obviously decreased aboveground net primary production (ANPP) relative to the normal rainfall pattern in a mesic grassland [1]. Thus, the pattern that describes how photosynthetic capacity is associated with soil moisture gradient might be complex, rather than the simple linear relationship only from a few drought treatments. In this study, we determined the changes of plant growth and photosynthetic capacity as plants were subjected to soil moisture gradients (soil relative water content 20%-90%). Our hypothesis was that the response pattern along a soil moisture gradient might show no simple linear form, and that plant growth and photosynthesis might increase with increasing water content within an intermediate soil moisture range, but decreasing with excessive soil moisture.

## Results

### Soil moisture changes and plant water status sensitivity to soil moisture

**Figure 1:**
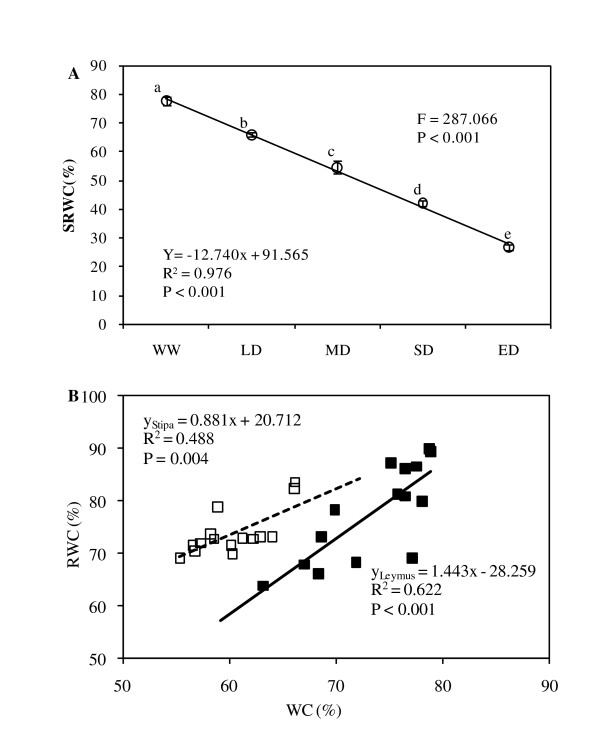
**Changes in soil relative water content (SRWC) as plants were subjected to the five levels of soil water-withholding treatments (A), and leaf relative water content (RWC) as a function of gravimetrical water content (WC) (B) for *L. chinensis *(filled squares, solid line) and *S. grandis *(open squares, dashed line).** Vertical bars in Figure 1A represent ±SE of the mean (n = 5) where these exceed the size of the symbol.

Water-withholding treatments led to a significant change in soil relative water content (SRWC, *F *= 287.066, *P *< 0.001), and a significant linear decrease in SRWC with decreased irrigation water amount (*Y *= -12.740× + 91.565), indicating water treatments can bring about a broad soil moisture gradient (Figure 1a). With regard to leaf relative water content (RWC) versus gravimetrical water content (WC) (expressed as percentage of fresh weight) in the same leaves, strong positive linear correlations were found (R^2 ^= 0.622, *P *< 0.001 for *L. chinensis*; and R^2 ^= 0.488, *P *= 0.004 for *S. grandis*) (Figure 1b), with a greater slope of the former, indicating a higher sensitivity in *L. chinensis *to soil drought.

### Plant growth response to soil moisture

**Figure 2:**
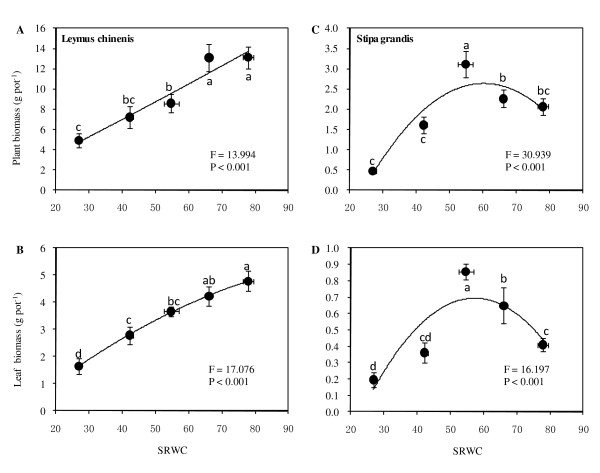
**Responses of plant (A, C) and leaf (B, D) biomass to soil relative water content (SRWC) for both *L. chinensis *(A, B) and *S. grandis *(C, D). **Vertical bars represent ±SE of the mean (n = 3-5) where these exceed the size of the symbol, and different lower case letters represent significant differences from water status according to Duncan's multiple range test.

A significant effect of soil moisture treatments on *L. chinensis *plant biomass was found (ANOVA: *F *= 13.99, *P *< 0.001) with significant reductions under moderate drought (MD), severe drought (SD), and extreme drought (ED), based on Duncan's multiple range test (*P *< 0.05). The relationship of plant biomass with SRWC showed an obviously typical pattern (Figure 2a): increasing sharply with soil moisture below approximately 66% of SRWC, and then leveling off when plant biomass peaked at a maximum of 13.1 g pot^-1^. The similar relationship between leaf biomass and SRWC appeared, with a maximum of 3.1 g pot^-1 ^(Figure 2b). Soil water significantly affected *S. grandis *plant and leaf biomass with significant enhancement as plants were subjected to light drought (LD, Figure 2c, d; *P *< 0.05), and the relationships of individual plant and leaf biomass with SRWC demonstrated similar response patterns. Plant and leaf biomass maxima were 3.1 and 0.86 g pot^-1 ^at 54.7% of SRWC, indicating a lower productivity for *S. grandis *relative to *L. chinensis *under moderate soil moisture.

### Light-response-curve photosynthetic potentials with regard to soil moisture

**Figure 3:**
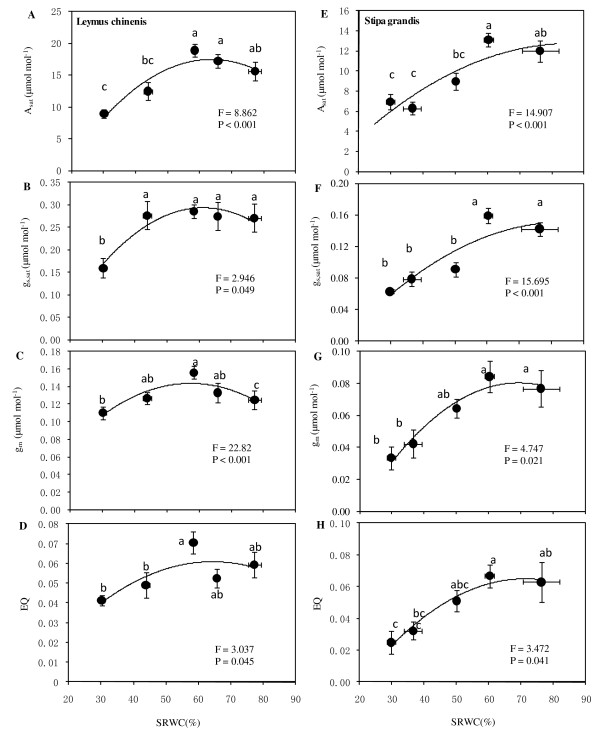
**Responses of light-saturated net CO**_**2 **_**assimilation rate (*A***_**sat**_**, A, E), light-saturated stomatal conductance (*g***_**s,sat**_**, B, F), mesophyll conductance (*g***_**m**_**, C, G), and maximum apparent quantum yield (*EQ*, D, H) in *L. chinensis *(A-D) and *S. grandis *(E-H).** Vertical bars represent ±SE of the mean (n = 3-5) where these exceed the size of the symbol. Note different scales and units in y-axes. Different lower case letters represent significant differences from water status according to Duncan's multiple range test.

The response of light-saturated net CO_2 _assimilation rate (*A*_sat_) in *L. chinensis *leaves to soil moisture was significant based on ANOVA (*F *= 8.862, *P *< 0.001) with significant decline as plants were exposed to drought stress below 50% SRWC (*P *< 0.05, Figure 3a). A maximum of 18.9 μmol mol^-1 ^occurred at 58.4% SRWC with a decrease thereafter, which may represent a soil moisture optimum threshold for maximizing photosynthetic potential.

Soil moisture treatments also produced significant changes in light-saturated stomatal conductance (*g*_s,sat_) (*F *= 2.946, *P *= 0.049), and extreme drought demonstrated a significant decline relative to other water treatments (*P *<.05, Figure 3b). A flat horizontal line on response curve of correlation between *g*_s,sat _and SRWC was also observed above 58.4% of SRWC, with a *g*_s,sat _peak of 0.29 mol mol^-1^. Additionally, under soil water treatments, significant changes in mesophyll conductance (*g*_m_) occurred (*F *= 22.82, *P *< 0.001), with a pattern similar to *g*_s,sat _in response to water status (Figure 3c). The maximum apparent quantum yield of CO_2 _uptake (*EQ*) significantly changed under water treatments (*F *= 3.037, *P *= 0.045) with a significant ED-induced decline (Figure 3d). The flat horizontal line of the response appeared at the same SRWC level, with *EQ *peaking at 0.071 (dimensionless).

For *S. grandis*, soil water treatments led to significant changes in the three key photosynthetic potential parameters derived from the light-response curves (*P *< 0.05, Figures 3e-h). A similar pattern concerning the relationship between *A*_sat _and SRWC was obtained, with a peak *A*_sat _of 13.1 μmol mol^-1 ^versus 60.3% SRWC (Figure 3e). For the relationships between *g*_s,sat_, *g*_m_, and *Q *with SRWC, the maxima were 0.16 mol mol^-1^, 0.084 mol mol^-1^, and 0.067 at the same SRWC of 60.3% (Figures 3f-h). The results also indicated *S. grandis *had a higher photosynthetic capacity under a proper water status.

### *A*/*C*_i _curve photosynthetic potentials with regard to soil moisture

**Figure 4:**
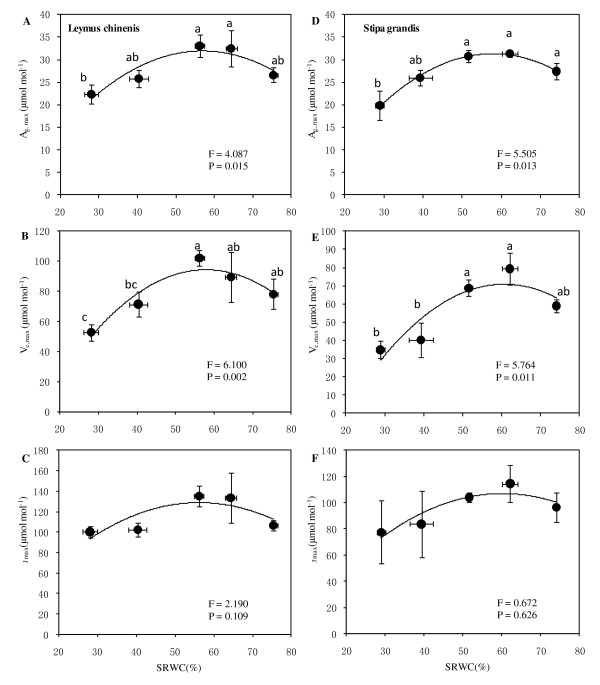
**Responses of maximum gross CO**_**2 **_**assimilation rate (*A***_**g,maxt**_**, A, D), maximum carboxylation velocity (*V***_**c,max**_**, B, E), and maximum rate of electron transport (*J***_**max**_**, C, F) in *L. chinensis *(A-C) and *S. grandis *(D-F).** Vertical bars represent ±SE of the mean (n = 3-5) where these exceed the size of the symbol. Note different scales and units in y-axes. Different lower case letters represent significant differences from water status according to Duncan's multiple range test.

For *L. chinensis *plants, there were significant effects on maximum gross CO_2 _assimilation rate (*A*_g,max_) and maximum carboxylation velocity (*V*_c,max_) due to the water treatments. The response curves with the flat plateaus were also obtained with *A*_g,max _and *V*_c,max_, but no significant effect of soil moisture on the maximum rate of electron transport (*J*_max_) was observed (*P *>.05) (Figures 4a-c). Overall, the maxima of *A*_g,max_, *V*_c,max_, and *J*_max _were 33.1, 102.2, and 135.3 μmol mol^-1^, respectively, within a similar SRWC range, approximately 56.1-64.3%.

For *S. grandis*, the responses of *A*_g,max _and *V*_c,max _but not *J*_max _to soil water treatments were significant (*P *< 0.05) (Figures 4d-f). The relationships of the former two parameters with SRWC again demonstrated similar patterns to those of *L. chinensis*, while the relationship of *J*_max _with SRWC was weak (*P *= 0.626). The maxima of the three critical parameters' responses to soil moisture were 31.2, 79.4, and 114.5 μmol mol^-1^, respectively, at a SRWC level of 66.1%.

### PSII photochemical potentials with regard to soil moisture

**Figure 5:**
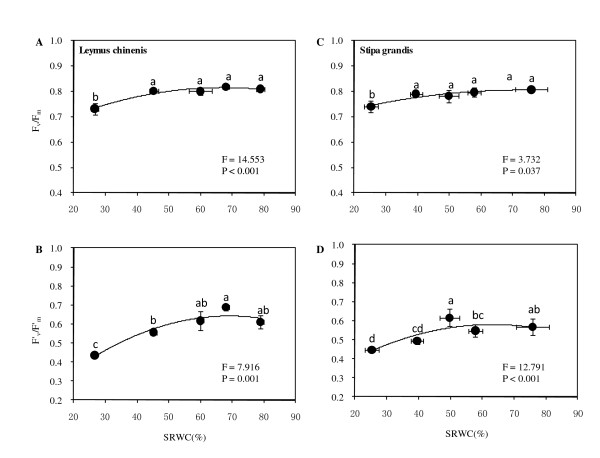
**Responses of *F***_**v**_**/*F***_**m **_**(A, C) and *F'***_**v**_**/*F'***_**m **_**(B, D) to soil relative water content (SRWC) for both *L. chinensis *(A, B) and *S. grandis *(C, D).** Vertical bars represent ±SE of the mean (n = 3-5) where these exceed the size of the symbol. Different lower case letters represent significant differences from water status according to Duncan's multiple range test.

Changes in soil moisture resulted in significant alterations to the maximal efficiency of PSII photochemistry (*F*_v_/*F*_m_) and efficiency of excitation energy capture by open PSII reaction centers (*F'*_v_/*F'*_m_) for both grass species (ANOVA, all, *P *< 0.037, Figures 5a-d) with significant decreases under ED stress (*P *< 0.05). Generally, for *L. chinensis*, no significant effects of water moisture above the 60.0% level of SRWC were found in respect of *F*_v_/*F*_m _and *F'*_v_/*F'*_m _(Figures 5a and b). At a level of 68.0% SRWC, the maxima for *F*_v_/*F*_m _and *F'*_v_/*F'*_m _were 0.82 and 0.68, respectively. For *S. grand*is, analogous patterns were obtained: *F*_v_/*F*_m _peaked at 0.80 (SRWC 57.8%), and *F'*_v_/*F'*_m _peaked at 0.62 (SRWC 50.0%) (Figures 5c, d).

## Discussion

Water scarcity is a central issue constraining productivity in most grassland ecosystems [12,29,30,35], and can drastically affect photosynthesis at various levels, from molecular through biochemical/physiological to individual aspects [3,15,22]. Different photosynthetic parameters can demonstrate different irregular changes in response to drought, involving interconnections and tradeoffs of great complexity, which strongly depend on the species [22,36-38]. In the present study, which utilized a broad range of soil water status, the responses of plant functions including growth and photosynthetic potential to soil water status, showed the patterns with photosynthetic capacity peaks at moderate soil water availability but declines under severe drought and excessive moisture, as well as differences between a perennial rhizome grass and a bunchgrass.

Severe drought leads to great reduction and irreversible damage of photosynthesis, due to both diffusion limitations (mainly decreases in stomatal and mesophyll conductance) and biochemical limitations (e.g., decreases in *V*_c,max _and *F*_v_/*F*_m_), the latter becoming more critical with drought intensity [3,13,19,39]. On the other hand, excess water in soil can also result in a drastic reduction of photosynthesis [33,40,41]. As excessive water in soil existed, plant malfunctions, such as disturbance in hormone signals, oxidative damage, and the accumulation of toxic products of anaerobic metabolism due to anoxia in the rhizosphere, can occur [42,43], as well as decreases in phloem transport [34,44]. Together these may correlatively negatively affect photosynthesis, stomatal conductance, and PSII functionality.

In a larger regional geographic transect, the results of Jiang and Dong [45] indicated that net photosynthetic rate (*A*) appears to have high points in the middle region along the Northeast China Transect, with an annual precipitation ranging from 177 mm in the east to 706 mm in the west. This implies the relationship between gas exchange and water availability on a large spatial scale. However, within a wide range of high precipitation (1800-3500 mm year^-1^) in lowland Panamanian forest, leaf photosynthesis decreases rather than increases with increasing precipitation [46], indicating that in the wetter region, excessive moisture may limit photosynthetic activity.

**Figure 6:**
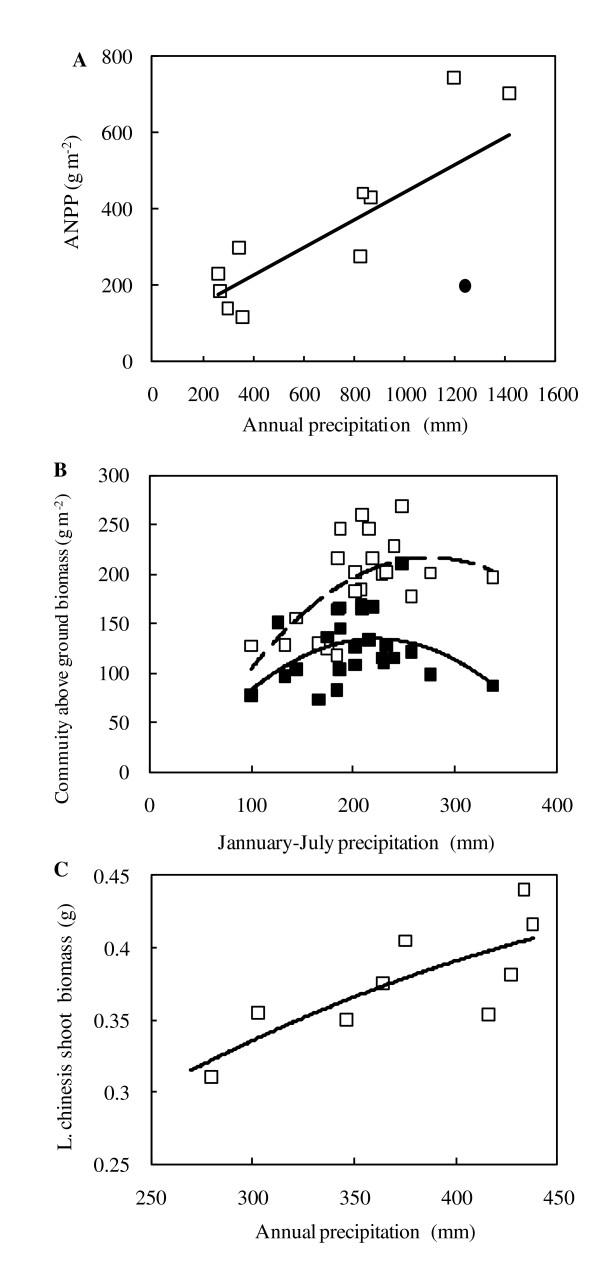
**Relationship of average annual aboveground net primary production (ANPP) with average annual precipitation at a continental scale (A, a filled circle represents the point of the lowest ANPP with high precipitation at the meadow site) [data from Knapp and Smith **[47]]; **relationship of community aboveground biomass with January-July precipitation in *L. chinensis *site (B, open squares, dashed line) and *S. grandis *site (filled squares, solid line) in the Inner Mongolia grassland [data from Bai et al. **[2]]; **and relationship of *L. chinensis *shoot biomass and annual precipitation along the Northeast China Transect, a precipitation gradient [C, data from Wang and Gao **[8]].

At the ecosystem or community levels, the specific relationships of grassland productivity and growth functions with water status including precipitation gradient are receiving more and more attention. On a continental scale, Knapp and Smith [47] indicated that ANPP has a strong linear relationship with annual precipitation, but there is an exceptional point of the lowest ANPP with the highest precipitation at the moist alpine meadow site (Figure 6a). While Bai et al. [2] reported a significant linear relationship between *L. chinensis *community biomass and temporal precipitation, a response curve with a flat plateau also could be obtained as reanalyzed (Figure 6b). After further analyzing the report by Wang and Gao [8], we also obtained a similar relationship between annual *L. chinensis *shoot biomass and a spatial precipitation gradient along a relatively large regional transect (Figure 6c). According to the recent report by Bai et al. [9], ANPP linearly increases with elevating mean annual precipitation (MAP) at both spatial and temporal scales. Regarding the relationship between relative biomass and MAP, they [9] indicated that a unimodal relationship appeared between the mean relative biomass of perennial grasses and MAP across different sites. However, for perennial forbs, a positive linear correlation was found, suggesting that the form of ecosystem composition shift due to the changes in MAP may depend on the plant functional group (PFG). By the same token, the response of grassland productivity to grazing pressures and/or unappreciated land use might also demonstrate the relationship pattern with a threshold shift. For instance, Sasaki et al. [48] found the evidence that the presence of a threshold in vegetation changed in response to a grazing gradient in the Mongolian rangelands, indicating that the productivity of grassland can be favored under moderate grazing pressure. In contrast, a deleterious turning point appeared under an extreme high grazing level. In summary, we have obtained strong evidence confirming the hypothesis of the presence of discontinuities rather than simple linear relationships under a large range of environmental gradients (such as water status) and land use intensification (such as grazing level), from the physiologically photosynthetic leaf to ecosystem levels.

## Conclusions

Based on our study results and the reanalysis on other relevant reports cited above, the response curves with flat plateaus may be found often in most of the responses of plant functional capacity to an environmentally variable gradient; although the curves concerning the response pattern are not always well justified due to a few exceptional points appearing, that needs further studies in detail. Species with different photosynthetic response patterns to water content can also partly explain the issues concerning the invasion of the grasslands by exotic species [49]. For example, those grasses with a high photosynthetic regulating ability to soil moisture, which are introduced, are always successful in regard to opportunity of invasion [36], and some climatic extremes, including severe drought, may determine the ecological success of grasses with different photosynthetic pathways [35,37]. Generally, the community dominated by *S*. *grandis *is more drought-resistant than that dominated by *L. chinensis*, and the former is often distributed in more arid areas [50,51]. In the present experiments, *S*. *grandis *was found to have lower productivity and photosynthetic potentials, but had generally similar patterns of the relationship between photosynthetic potentials and water status relative to *L. chinensis*. Thus, the present results showing the threshold presences in photosynthetic capacity in response to a broader range of soil moisture may also provide a new insight into species component dynamics at an ecosystem level in the changing environment.

## Methods

### Seed collection and plant culture

Steppe grasslands dominated by *Leymus chinensis *(Trin.) Tzvel and *Stipa grandis *P. Smirn are the two key vegetation types that widely cover the largest contiguous natural grassland region of the world, from the Eastern Eurasian steppe (semi-arid and sub-humid) to the middle Eurasian steppe zone (semi-arid). The former species represents a native, clonal perennial rhizomatic grass, while the latter is a perennial bunchgrass [8,9]. Both species can provide good livestock forage, and co-occur mainly in the natural grazing rangeland.

In the last year of our studies, the seeds were obtained from grassland in Xilinhot, Inner Mongolia, China (44°08' N, 117°05' E), 1100 m above sea level. This region is a semiarid grassland that experiences a continental climate with mild temperatures during spring and autumn, cool and dry winters, and wet but hot summers. Annual mean temperature and precipitation were 2°C and 350 mm over the last 50 years, respectively.

Seeds of *L. chinensis *and *S. grandis *were sterilized with 0.7% potassium permanganate solution for 8 minutes, and rinsed before transfer into a refrigerator below 0°C for 7 d. They were sown in plastic pots (5.1 L, 18 cm in diameter, 20 cm in height) wrapped with plastic film to avoid drainage. Each pot was filled with 4.08 kg of dry soil obtained from the local field in which the seeds were collected, with a density of 4 *L. chinensis *and 4 *S. grandis *plants. In the chestnut-coloured soil, the organic carbon and total nitrogen concentrations were 19.60 ± 0.18 g kg^-1 ^and 4.18 ± 0.11 g kg^-1^, respectively. Soil field capacity was 29.3%. All experimental pots were placed in a naturally illuminated greenhouse at a daily maximum photosynthetic photon flux density (*PPFD*) of approximately 1000 μmol m^-2 ^s^-1 ^above the plant canopy. Illumination was provided by a combination of cool-white fluorescent and incandescent lamps within the greenhouse, with a day/night temperature of 26-28/18-20°C.

Soil moisture levels were maintained using manual irrigation by weighing individual pots at 5:00 pm daily. Each target of the desired soil moisture range was achieved by decreasing the water supply progressively over a period of about 20 d. To obtain a relatively stable water moisture gradient, soil relative water content (SRWC), i.e., the ratio between present soil moisture and field capacity, was divided into 5 levels: well-watered (WW, 70-80%), light drought (LD, 60-70%), moderate drought (MD, 50-60%), severe drought (SD, 35-50%), and extreme drought (ED, 25-30%), respectively. The measurement was made 56-60 d after the plants had been subjected to the relative long-term soil water treatments. Each group (SRWC level) had at least 10 pots as replicates. The arrangements of pots with different treatments were randomized daily to avoid effects from other environmental factors, such as light and temperature conditions.

### Leaf relative water content (RWC)

Detached leaves (0.5 g fresh weight) were cut and weighed immediately to obtain fresh weight (FW), and were then placed in a beaker filled with water overnight in the dark to obtain turgid fresh weight (TW) the next morning. Dry weight (DW) was obtained after drying at 80°C for at least 24 h in an oven. The relative water content (RWC) of the leaves was expressed as RWC = [(FW - DW)/(TW - DW)] ×100; water content (WC) was calculated as WC = (FW - DW)/FW ×100.

### Leaf gas exchange and chlorophyll fluorescence measurements

Combined leaf gas exchange and chlorophyll fluorescence measurements were conducted using an open gas exchange system (LI-6400; LI-COR, Inc., Lincoln, NE, USA) with a leaf chamber fluorometer attachment (LI-6400-40). Illumination was supplied to leaves from a red-blue LED light source and data initially were analyzed with data acquisition software (OPEN 5.1, LI-COR). Before making measurements, leaves were acclimated in the chamber for at least 10 minutes at 25°C with an ambient CO_2 _concentration of 380 μmol mol^-1 ^and a *PPFD *of 900 μmol m^-2^s^-1^, conditions under which photosynthesis is nearly saturated. Determinations of gas exchange parameters were made on at least 3 the youngest and fully expanded leaves from different individuals (1 plant per pot) for all replicates, 8:30 to 15:30 h daily. The vapour pressure deficit (VPD) in the cuvette was maintained below 2.0 kPa.

The measurements for fluorescence parameters were conducted simultaneously on the same leaves for gas exchange determination after 30 minutes of dark adaptation at 25°C. The minimal fluorescence yield (*F*_o_) was measured by using modulated light that was sufficiently low (< 0.1 μmol m^-2^s^-1^), and the maximal fluorescence yield (*F*_m_) was determined by a 0.8 s saturating pulse at 8000 μmol m^-2^s^-1 ^in the dark-adapted leaves. Leaves were then continuously illuminated with white actinic light at an intensity of 900 μmol m^-2^s^-1 ^for 20 minutes. The steady-state value of fluorescence (*F*_s_) was thereafter recorded and the second saturating pulse at 8000 μmol m^-2^s^-1 ^was imposed to determine the maximal light-adapted (*F'*_m_) fluorescence level. The actinic light was removed and the minimal fluorescence level in the light-adapted state (*F'*_0_) was determined after 3 s of far-red illumination. The fluorescence parameters were obtained from the formulae [52]: maximal efficiency of photosystem II (PSII) photochemistry *F*_v_/*F*_m _= (*F*_m _- *F*_0_)/*F*_m_, efficiency of excitation energy captured by open PSII reaction centers *F'*_v_/*F'*_m _= (*F'*_m _- *F'*_0_)/*F'*_m_, and the actual PSII efficiency Φ_PSII _= (*F'*_m _- *F*_s_)/*F'*_m_.

### Estimation of light response parameters

After acclimation, the *PPFD *was sequentially lowered to 1200, 900, 800, 600, 400, 200, 100, 50, and 20 μmol m^-2^s^-1^. The response parameters of photosynthesis to light were estimated by a quadratic equation devised by Long et al. [53]:

$$A=\left(EQ*PPFD+{\left({\left(EQ*PPFD+A_{\text{sat}}\right)}^{\text{2}}–\text{4}EQ*PPFD*\theta *A_{\text{sat}}\right)}^{0.\text{5}}\right)\;/\left(\text{2}\theta \right)-R_{\text{d}}$$

where *A *is net photosynthetic rate (μmol m^-2^s^-1^), *A*_sat _the light-saturated CO_2 _accumulation rate (μmol m^-2 ^s^-1^), *PPFD *the photosynthetic photon flux density (μmol m^-2 ^s^-1^), *EQ *leaf maximum apparent quantum yield of CO_2 _uptake, *θ *the convexity of the transit from light-limited to light-saturated photosynthesis, and *R*_d _the respiration rate in the dark. Light-saturated stomatal conductance (*g*_s,sat_) was obtained after illuminating with saturating light (900 μmol photon m^-2 ^s^-1^) for at least 10 minutes at 25°C. *R*_d _was measured after dark conditions of at least 15 min.

### Estimation of photosynthesis response to *C*_i_

A modified photosynthetic model was used to analyze the *A*/*C*_i _response curve to obtain key photosynthetic capacity parameters; thereafter the relationship of these parameters with soil moisture was conducted using a curve estimation analysis. The CO_2 _concentration gradient for the *A*/*C*_i _curves was 380, 300, 200, 100, 50, 20, 380, 380, 600, 800, and 1000 μmol m^-2^s^-1^, step by step. To obtain *V*_c,max_, *J*_max_, and *A*_g,max_, a modified curve-fitting software was used to analyze the *A*/*C*_i _responses reported by Sharkey et al. [54] based on the original model of Farquhar et al. [28], except that mesophyll conductance (*g*_m_) was obtained from the Harley et al. [55] equation in which the value for *g*_m _was fixed when adjusting the *A*/*C*_i _curves using procedure of Sharkey et al. [54]: *g*_m _= *A*/(*C*_i _- Г*(*J *+ 8(*A *+ *R*_d_))/(*J *- 4(*A *+ *R*_d_))).

The electron transport rate (*J*) was expressed as *J *=Φ_PSII _× *fI*α_leaf_, where *I *is actinic *PPFD*; *f *is the fraction of absorbed quanta that is used by PS II, and is typically assumed to be 0.5. The *α*_leaf _is effective leaf absorbance, ranging between 0.88 and 0.95 in different species from the data measured by Flexas et al. [56]. Due to experimental facility limitation, many experimental studies have not measured the value, which is assumed to be 0.85 [19]. For our experiments, we assumed a middle value of 0.88. Φ_PSII _was directly measured using a leaf chamber fluorometer attachment (LI-6400-40 LCF) and the formulae of van Kooten and Snel (1990) [52]. *R*_d _was measured after dark conditions of at least 15 min.

### Biomass

For the biomass measurements, samples of 5 pots from each treatment, separated into leaves and other parts, were immediately placed in a dryer, and dried at 80°**C **to constant weight to obtain dry matter.

### Data analysis

All statistical analysis was performed using SPSS 17.0 (SPSS, Chicago, Illinois, USA). Effects of soil moisture treatments on plant biomass, leaf biomass, and photosynthetic parameters were conducted by ANOVA; when the effects were significant (*P *< 0.05), Duncan's multiple range test was used to compare soil water treatments (*P *< 0.05). Key parameters were estimated using curve estimations and nonlinear regression. Statistic significance for all analyses was the 0.05 probability level unless stated otherwise.

## Abbreviations

*A*_g,max_: maximum gross CO_2 _assimilation rate; *A*_sat_: light-saturated net CO_2 _assimilation rate; *EQ*: maximum apparent quantum yield of CO_2 _uptake; *F*_v_/*F*_m_: maximal efficiency of PSII photochemistry; *F'*_v_/*F'*_m_: efficiency of excitation energy capture by open PSII reaction centers_; _*g*_s_: stomatal conductance; *g*_m_: mesophyll conductance; *g*_s,sat_: light-saturated *g*_s_; *J*_max_: maximum rate of electron transport; *V*_c,max_: maximum *in vivo *carboxylation velocity.

## Authors' contributions

ZX designed and conducted the experiments, and wrote the manuscript. GZ directed the study and reviewed the manuscript. All authors have read, discussed, and approved the final manuscript.
